# Highly Effective Therapies as First-Line Treatment for Pediatric-Onset Multiple Sclerosis

**DOI:** 10.1001/jamaneurol.2023.5566

**Published:** 2024-02-12

**Authors:** Nail Benallegue, Fabien Rollot, Sandrine Wiertlewski, Romain Casey, Marc Debouverie, Anne Kerbrat, Jérôme De Seze, Jonathan Ciron, Aurelie Ruet, Pierre Labauge, Elisabeth Maillart, Helene Zephir, Caroline Papeix, Gilles Defer, Christine Lebrun-Frenay, Thibault Moreau, Eric Berger, Bruno Stankoff, Pierre Clavelou, Olivier Heinzlef, Jean Pelletier, Eric Thouvenot, Abdullatif Al Khedr, Bertrand Bourre, Olivier Casez, Philippe Cabre, Abir Wahab, Laurent Magy, Sandra Vukusic, David-Axel Laplaud

**Affiliations:** 1 Department of Paediatric Neurology, Universitaire Angers, CHU Angers, Angers, France; 2 Nantes Université, CHU Nantes, Inserm, CIC 14131413, Center for Research in Transplantation and Translational Immunology, UMR 1064, Nantes, France; 3 Université de Lyon, Université Claude Bernard, Lyon 1, Lyon, France; 4 Department of Neurology, Hôpital Neurologique Pierre Wertheimer, Hospices Civils de Lyon, Sclérose en Plaques, Pathologies de la Myéline et Neuro-Infammation, Bron, France; 5 Centre de Recherche en Neurosciences de Lyon, Observatoire Français de La Sclérose en Plaques, Inserm 1028 et CNRS UMR 5292, Lyon, France; 6 EUGENE DEVIC EDMUS Foundation Against Multiple Sclerosis, State-Approved Foundation, Bron, France; 7 Department of Neurology, CHU Nantes, Nantes, France; 8 Department of Neurology, Centre Hospitalier Régional Et Universitaire de Nancy, Université de Lorraine, 4360 APEMAC Vandoeuvre-Lès-Nancy, EA, France; 9 Rennes University, CHU Rennes, CRC-SEP Neurology Department, and EMPENN U 1228, Inserm, INRIA, CNRS, Rennes, France; 10 Department of Neurology Et Centre d’Investigation Clinique, CHU de Strasbourg, INSERM 1434, Strasbourg, France; 11 Department of Neurology, CRC-SEP, CHU de Toulouse, Hôpital Pierre-Paul Riquet, Toulouse, France; 12 Institut Toulousain Des Maladies Infectieuses Et Inflammatoires (Infinity), Inserm UMR 1291, CNRS UMR 5051, Université Toulouse III, Toulouse, France; 13 Department of Neurology, CHU de Bordeaux, Bordeaux, France; 14 Université de Bordeaux, Inserm, Neurocentre Magendie, U1215 Bordeaux, France; 15 CRC SEP, Department of Neurology, Montpellier Universitary Hospital, Montpellier, France; 16 Department of Neurology, APHP, Hôpital Pitié-Salpêtrière, Paris, France; 17 Pôle Des Neurosciences Et de L’appareil Locomoteur, CRC-SEP, Hôpital Roger Salengro, Université de Lille, Inserm U1172, Lille, France; 18 Département of Neurology, Hôpital Fondation A.de Rothschild, Paris, France; 19 Department of Neurology, Centre Expert SEP, CHU de Caen, Université Normandie, Caen, France; 20 CRC-SEP Neurologie Pasteur 2, CHU de Nice, Université Cote d’Azur, UMR2CA (URRIS), Nice, France; 21 Department of Neurology, CHU de Dijon, Dijon, France; 22 Department of Neurology, CHU de Besançon, Besançon, France; 23 Department of Neurology, CHU Saint-Antoine, Paris, France; 24 Department of Neurology, CHU de Clermont-Ferrand, Clermont-Ferrand, France; 25 Département de Neurologie, Centre Hospitalier de Poissy, St Germain, France; 26 Aix Marseille Univ, APHM, Hôpital de la Timone, Pôle de Neurosciences Cliniques, Service de Neurologie – MICeME, CRMBM CEMEREM UMR7339, Marseille, France; 27 Department of Neurology, CHU de Nîmes, Nîmes, France; 28 IGF, University Montpellier, CNRS, Inserm, Montpellier, France; 29 Department of Neurology, CHU d’Amiens, Amiens, France; 30 CHU Rouen, Rouen, France; 31 Department of Neurology, CHU de Grenoble, Grenoble, France; 32 Department of Neurology, CHU de Fort de France, Fort de France, France; 33 Department of Neurology, Assistance Publique des Hôpitaux de Paris, Hôpital Henri Mondor, Université Paris Est, Créteil, France; 34 Department of Neurology, CHU de Limoges, Limoges, France

## Abstract

**Importance:**

Moderately effective therapies (METs) have been the main treatment in pediatric-onset multiple sclerosis (POMS) for years. Despite the expanding use of highly effective therapies (HETs), treatment strategies for POMS still lack consensus.

**Objective:**

To assess the real-world association of HET as an index treatment compared with MET with disease activity.

**Design, Setting, and Participants:**

This was a retrospective cohort study conducted from January 1, 2010, to December 8, 2022, until the last recorded visit. The median follow-up was 5.8 years. A total of 36 French MS centers participated in the Observatoire Français de la Sclérose en Plaques (OFSEP) cohort. Of the total participants in OFSEP, only treatment-naive children with relapsing-remitting POMS who received a first HET or MET before adulthood and at least 1 follow-up clinical visit were included in the study. All eligible participants were included in the study, and none declined to participate.

**Exposure:**

HET or MET at treatment initiation.

**Main Outcomes and Measures:**

The primary outcome was the time to first relapse after treatment. Secondary outcomes were annualized relapse rate (ARR), magnetic resonance imaging (MRI) activity, time to Expanded Disability Status Scale (EDSS) progression, tertiary education attainment, and treatment safety/tolerability. An adapted statistical method was used to model the logarithm of event rate by penalized splines of time, allowing adjustment for effects of covariates that is sensitive to nonlinearity and interactions.

**Results:**

Of the 3841 children (5.2% of 74 367 total participants in OFSEP), 530 patients (mean [SD] age, 16.0 [1.8] years; 364 female [68.7%]) were included in the study. In study patients, both treatment strategies were associated with a reduced risk of first relapse within the first 2 years. HET dampened disease activity with a 54% reduction in first relapse risk (adjusted hazard ratio [HR], 0.46; 95% CI, 0.31-0.67; *P* < .001) sustained over 5 years, confirmed on MRI activity (adjusted odds ratio [OR], 0.34; 95% CI, 0.18-0.66; *P* = .001), and with a better tolerability pattern than MET. The risk of discontinuation at 2 years was 6 times higher with MET (HR, 5.97; 95% CI, 2.92-12.20). The primary reasons for treatment discontinuation were lack of efficacy and intolerance. Index treatment was not associated with EDSS progression or tertiary education attainment (adjusted OR, 0.51; 95% CI, 0.24-1.10; *P* = .09).

**Conclusions and Relevance:**

Results of this cohort study suggest that compared with MET, initial HET in POMS was associated with a reduction in the risk of first relapse with an optimal outcome within the first 2 years and was associated with a lower rate of treatment switching and a better midterm tolerance in children. These findings suggest prioritizing initial HET in POMS, although long-term safety studies are needed.

## Key Points

**Question:**

What is the optimal initial treatment strategy in pediatric-onset multiple sclerosis to control disease activity?

**Findings:**

In this multicenter cohort study that included 530 children, those who started taking highly effective therapies had an associated 54% lower risk of first relapse at 5 years than those taking moderately effective therapies.

**Meaning:**

Results suggest that in pediatric-onset multiple sclerosis, initiating highly effective rather than moderately effective therapies may better control early disease activity.

## Teaser

This cohort study investigates the optimal initial treatment strategy in pediatric-onset multiple sclerosis to control disease activity.

## Introduction

Treatments in pediatric-onset multiple sclerosis (POMS) have largely been used off-label until the recent approval of fingolimod, dimethyl fumarate (DMF), and teriflunomide. Fingolimod has demonstrated a marked superiority over interferon beta-1a in the 2-Year, Double-Blind, Randomized, Multicenter, Active-Controlled Core Phase to Evaluate Safety & Efficacy of Daily Fingolimod vs Weekly Interferon Beta-1a in Pediatric Patients With Multiple Sclerosis and 5 Year Fingolimod Extension Phase (PARADIGMS) randomized clinical trial (RCT) in reducing both clinical and magnetic resonance imaging (MRI) activity with a relative improvement in quality of life despite a slightly higher incidence of serious adverse events (SAEs).^1,2^ Teriflunomide and DMF were recently approved by the US Food and Drug Administration and European Medicine Agency following the phase 3 Efficacy, Safety, and Pharmacokinetics of Teriflunomide in Pediatric Patients With Relapsing Forms of Multiple Sclerosis (TERIKIDS) and Phase 3 Efficacy and Safety Study of BG00012 in Pediatric Subjects With Relapsing-Remitting Multiple Sclerosis (CONNECT) RCTs.^3,4,5^ These successes paved the way for other ongoing RCTs with highly potent drugs like ocrelizumab, ofatumumab, and siponimod.^6,7^

In the last decade, the availability of highly potent drugs led to a decrease in long-term disability accrual.^8^ Several observational studies have suggested the beneficial outcomes of newer and off-label disease-modifying therapies (DMTs) on clinical disease and radiological activity over interferons and glatiramer acetate.^8,9,10,11,12,13,14^

Early treatment after MS onset has shown benefits in reducing persistent disability in children.^8,15^ The highly inflammatory disease in children compared with adults suggests the need for prompt therapeutic control of the disease to prevent earlier disability, cognitive impairment, and brain volume loss.^8,12,15,16,17,18,19^ A common strategy in adult MS and POMS is to escalate treatment from moderately effective therapies (METs; ie, azathioprine, cyclophosphamide, dimethyl fumarate, glatiramer acetate, interferon beta, methotrexate, mycophenolate mofetil, teriflunomide) to highly effective therapies (HETs; ie, alemtuzumab, fingolimod, mitoxantrone, cladribine, natalizumab, ocrelizumab, ofatumumab, rituximab) in nonresponders.^20,21^ Yet, given the risk of disease breakthrough in POMS and putative long-term sequelae of first inflammatory events, assessing treatment strategy is critical.^16,22,23,24,25^ We used the national French MS cohort Observatoire Français de la Sclérose en Plaques (OFSEP) to address the effectiveness of index treatment strategy in POMS by evaluating clinical and radiological disease activity, disability, safety, and tolerance.

## Methods

### Study Design

This observational cohort study was approved by the Scientific Council of OFSEP and was based on data from 36 French expert centers participating in the French MS database OFSEP.^26^ For each patient, clinical and imaging data were collected during routine follow-up visits using a dedicated software, the European Database on Multiple Sclerosis (EDMUS [Eugene Devic EDMUS Foundation]), by a neurologist with a particular interest in MS.^27^ These data were collected retrospectively at the time of the first visit and prospectively thereafter at least once a year.

Patients enrolled in the OFSEP study^28^ provided written informed consent for participation. The OFSEP cohort was approved by both the French data protection agency and a French ethics committee. This study followed the Strengthening the Reporting of Observational Studies in Epidemiology (STROBE) reporting guidelines.

### Patients

Patients with POMS were included if they received a first treatment before the age of 18 years from January 1, 2010, to December 8, 2022, and if they were diagnosed with relapsing-remitting MS at treatment initiation (eMethods in Supplement 1).^29,30^ Eligible patients with POMS were categorized as starting with HET or MET. Information on participant race and ethnicity was not included in this study as collection of this information is banned by French law.

### Procedure

The therapies studied were administered according to published protocols (eMethods in Supplement 1). A treatment was considered interrupted if not assumed for more than 90 days for fingolimod, mitoxantrone, and natalizumab; 270 days for alemtuzumab, ocrelizumab, ofatumumab, and rituximab; and 30 days for all other treatments. Baseline was defined as treatment initiation, and patients with POMS were followed up until the last clinical evaluation.

### Outcomes

The primary outcome was the time to first relapse. Secondary outcomes included annualized relapse rate (ARR), relapse rate during each 3-month period, MRI activity at 2 years, time to 6-month confirmed disability progression (CDP) measured with the Expanded Disability Status Scale (EDSS), time to discontinuation of the first treatment, tertiary education attainment, and incidence rate of SAE. The CDP was defined as a 1-point increase in EDSS score (1.5 points if baseline EDSS score = 0 and 0.5 point if baseline EDSS score ≥5.5) sustained or increased over 6 months. MRI activity was defined by new T2 lesions compared with the index brain MRI scan or gadolinium-enhancing lesions.

### Statistical Analysis

We used an adapted statistical model with flexible parameters to estimate the dynamic event rate according to the time for a given patient. The cumulative proportion of patients for whom the identified event occurred is represented as an adjunctive statistical analysis. This approach facilitates data visualization, allowing the graphical representation of event dynamics for a given patient while providing smooth estimates of survival (eMethods in Supplement 1).^31,32,33^

For each time to event outcome, this framework was used to study the impact of the 2 treatment groups (HET vs MET). Then, confounding factors at baseline causing both the outcome (*P* value <.05) and the treatment group allocation (Cohen values *d* >0.2) were introduced in the model. The final model was selected based on the corrected Akaike information criterion (AIC), as a minimal AIC identifies the model that offers an optimal trade-off between the model’s goodness of fit and its parsimony.^34^

Regarding the secondary objectives (MRI activity and tertiary education level attainment, defined by enrollment in at least a short-cycle higher education program), a multivariate logistic regression was used by introducing associated factors causing both the outcome and the treatment group allocation and by providing the adjusted odds ratios (ORs) with their 95% CIs. For the former, only patients with POMS and at least a 2-year follow-up where MRI data available were included whereas for the latter, patients with POMS 20 years or older at the last visit were included to avoid selection bias. The estimations of relapse rate and 95% CI were estimated for the 12 months preceding HET or MET initiation and during a 24-month period postinitiation.

SAE incidence rates were defined as the total number of SAEs divided by the entire duration of the follow-up, and the 95% CIs were estimated assuming a Poisson distribution taking into account the exposure period of each treatment in patients with POMS whose treatments were switched after January 1, 2017, ie, the date of systematic collection of SAEs in the OFSEP cohort.

To evaluate the data using a more conventional statistical method, we used propensity scores by inverse probability of treatment weighting to compare the effectiveness of HET and MET on the occurrence of the first relapse after baseline in a sensitivity analysis.

The statistical analyses were conducted with an intention-to-treat approach, using SAS, version 9.4 (SAS Institute) for descriptive analyses and R software, version 4.2.2 (R Project for Statistical Computed) with the survPen package, version 1.0.0, to model event rate.^35^ All *P* values were 2-sided, and a *P* value <.05 was considered statistically significant.

## Results

### Baseline Demographic and Clinical Characteristics

**Figure 1.**
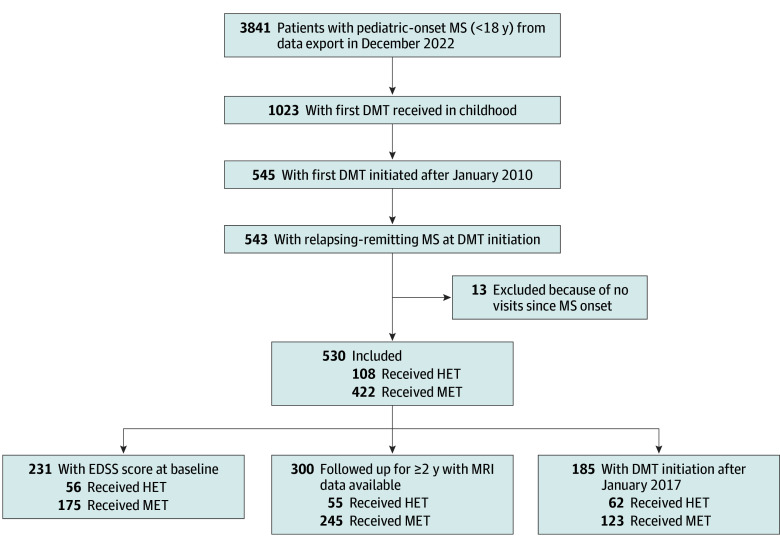
Study Flow Diagram DMT indicates disease-modifying therapy; HET, highly effective therapy; MET, moderately effective therapy; MS, multiple sclerosis.

In the OFSEP database, 74 367 patients with MS were recorded in December 2022, including 3841 (5.2%) with POMS. A total of 530 patients (mean [SD] age, 16.0 [1.8] years; 364 female [68.7%]; 166 male [31.3%]) with POMS having initiated a DMT between 2010 and 2022 were included in the present study (Table 1 and eTable 1 in Supplement1). METs were the most frequent index DMT used with 422 children (79.6%), whereas 108 children (20.4%) initiated HETs (Figure 1). The median (IQR) follow-up duration was 5.8 (3.0-8.7) years. Time from disease onset to baseline was similar in both groups (Table 1).

**Table 1.** Clinical and Radiological Baseline Characteristics of Eligible Children With Pediatric-Onset Multiple Sclerosis

Variable	No. (%)			Cohen *d*^a^
	Total	HET	MET	
No.	530	108 (20.4)	422 (79.6)	
Sex				
Male	166 (31.3)	36 (33.3)	130 (30.8)	0.05
Female	364 (68.7)	72 (66.7)	292 (69.2)	
Age at baseline, y				
<10	8 (1.5)	2 (1.9)	6 (1.4)	0.26
10-11	14 (2.6)	3 (2.8)	11 (2.6)	
12-13	42 (7.9)	4 (3.7)	38 (9.0)	
14-15	137 (25.9)	24 (22.2)	113 (26.8)	
16-17	329 (62.1)	75 (69.4)	254 (60.2)	
Treatment initiation epoch				
2010-2012	162 (30.6)	14 (13.0)	148 (35.1)	0.73
2013-2015	146 (27.6)	24 (22.2)	122 (28.9)	
2016-2018	120 (22.6)	27 (25.0)	93 (22.0)	
2019-2022	102 (19.3)	43 (39.8)	59 (14.0)	
EDSS (± 3 mo)				
Mean (SD)	1.2 (1.4)	2.2 (1.6)	0.8 (1.2)	0.95
Median (IQR) [range]	1.0 (0-2.0) [0.0-7.0]	2.0 (1.0-3.5) [0.0-6.5]	0.0 (0.0-1.5) [0.0-7.0]	
0.0	109 (20.6)	10 (9.3)	99 (23.5)	0.64
0.5-3.5	104 (19.6)	35 (32.4)	69 (16.4)	
≥ 4.0	18 (3.4)	11 (10.2)	7 (1.7)	
Not available	299 (56.4)	52 (48.2)	247 (58.5)	
Disease duration at first treatment, y				
Mean (SD)	1.1 (1.4)	0.9 (1.6)	1.1 (1.3)	0.12
Median (IQR) [range]	0.6 (0.3-1.2) [0-13.1]	0.6 (0.3-1.0) [0-13.1]	0.6 (0.3-1.3) [0-9.9]	
No. of relapses in the year before baseline				
0	11 (2.1)	3 (2.8)	8 (1.9)	0.42
1	257 (48.5)	35 (32.4)	222 (52.6)	
2	181 (34.1)	48 (44.4)	133 (31.5)	
≥3	81 (15.3)	22 (20.4)	59 (14.0)	
Brain MRI (−6 mo / +3 mo)				
Yes	389 (73.4)	88 (81.5)	301 (71.3)	0.24
No	141 (26.6)	20 (18.5)	121 (28.7)	
Gadolinium-enhancing brain lesions				
Positive	232 (59.6)	59 (67.0)	173 (57.5)	0.20
Negative	132 (33.9)	24 (27.3)	108 (35.9)	
Not available	25 (6.4)	5 (5.7)	20 (6.6)	
No. of T2 brain lesions				
0	2 (0.5)	0	2 (0.7)	0.43
< 9	57 (14.7)	6 (6.8)	51 (16.9)	
≥ 9	253 (65.0)	68 (77.3)	185 (61.5)	
Not available	77 (19.8)	14 (15.9)	63 (20.9)	
Brain MRI activity in the year before baseline				
Yes	341 (64.3)	73 (67.6)	268 (63.5)	0.06
No	49 (9.2)	12 (11.1)	37 (8.8)	
Not available	140 (26.4)	23 (21.3)	117 (27.7)	
**Index treatment**				
MET				
Azathioprine	6 (1.1)	0	6 (1.4)	NA
Cyclophosphamide	1 (0.2)	0	1 (0.2)	
Dimethyl fumarate	55 (10.4)	0	55 (13.0)	
Glatiramer acetate	58 (10.9)	0	58 (13.7)	
Interferon beta-1a	248 (46.8)	0	248 (58.8)	
Interferon beta-1b	11 (2.1)	0	11 (2.6)	
Peginterferon beta-1a	9 (1.7)	0	9 (2.1)	
Methotrexate	1 (0.2)	0	1 (0.2)	
Mycophenolate mofetil	1 (0.2)	0	1 (0.2)	
Teriflunomide	32 (6.0)	0	32 (7.6)	
HET				
Alemtuzumab	1 (0.2)	1 (0.9)	0	NA
Fingolimod	36 (6.8)	36 (33.3)	0	
Mitoxantrone	13 (2.4)	13 (12.0)	0	
Natalizumab	46 (8.7)	46 (42.6)	0	
Ocrelizumab	4 (0.8)	4 (3.7)	0	
Ofatumumab	1 (0.2)	1 (0.9)	0	
Rituximab	7 (1.3)	7 (6.5)	0	

Abbreviations: HET, highly effective therapy; MET, moderately effective therapy; NA, not applicable.

^a^ Standardized mean or proportion difference (Cohen *d* values): a value less than 0.2 is considered acceptable, between 0.2 and 0.5 as a moderate difference, between 0.5 and 0.8 as a significant difference, and greater than 0.8 as a major difference.

At baseline, treatment groups differed in relapse activity (≥3 relapses: HET, 22 of 108 [20.4%] vs MET, 59 of 422 [14.0%]) and disability (mean [SD] EDSS: HET, 2.2 [1.6] vs MET, 0.8 [1.2]; Cohen *d* >0.20). Patients taking an HET had a higher T2 lesion burden (≥9 brain lesions: HET, 68 of 108 [77.3%] vs MET, 185 of 422 [61.5%]) and appeared to have a higher percentage of gadolinium enhancement (positive enhancement: HET, 59 of 108 [67.0%] vs MET, 173 of 422 [57.5%]). HET mainly comprised natalizumab (46 of 108 [42.6%]) and fingolimod (36 of 108 [33.3%]), whereas interferon (268 of 422 [63.5%]), glatiramer acetate (58 of 422 [13.7%]), and DMF (55 of 422 [13%]) accounted for most MET. The enrollment of children taking an HET increased in the most recent epochs, after 2016 (eg, 2010-2012, 14 of 108 [13.0%] vs 2019-2022, 43 of 108 [39.8%]) (Table 1).

### Relapse Activity

**Figure 2.**
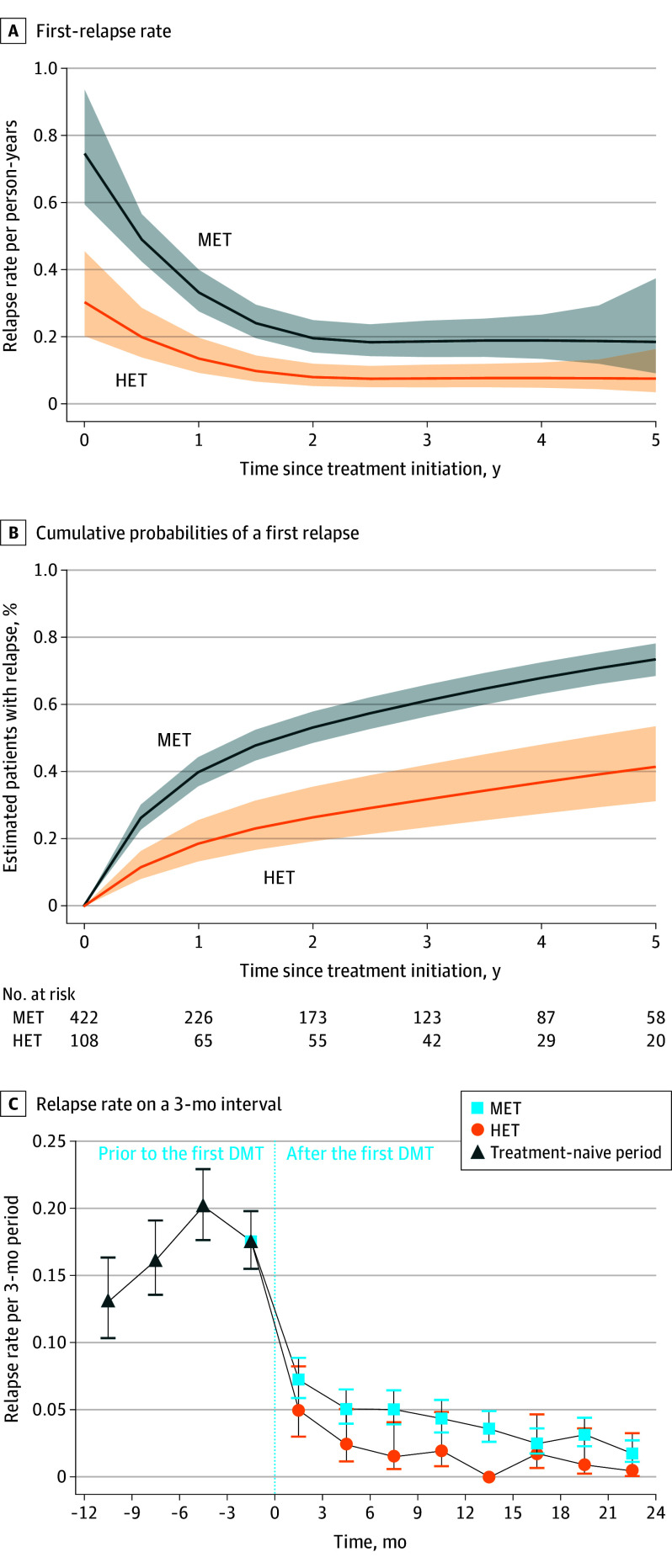
Relapse Rate According to Index Treatment Strategy A, First relapse rate. B, Cumulative probabilities of a first relapse occurrence after treatment initiation according to index disease-modifying therapy. C, Relapse rate on a 3-month interval basis, 12 months before and 24 months after disease-modifying therapy initiation according to first treatment strategy (95% CI). HET indicates highly effective therapy; MET, moderately effective therapy.

The number of events for primary and secondary outcomes are summarized in eTable 2 in Supplement 1. For the primary analysis, the model-building strategy, including flexible effects on the treatment variable, is shown in eTable 3 and eTable 4 in Supplement 1. There was a significant proportional treatment impact, ie, the observed outcome remained similar during follow-up. Both treatment strategies impacted the first relapse rate within the first 2 years, an outcome that was more pronounced for HET; then, the outcome was sustained (Figure 2A). Thus, from 2 years of follow-up, the probability of a first relapse was approximately 8% (8 relapses per 100 person-years) and 20% (20 relapses per 100 person-years) per year in the HET and MET groups, respectively (Figure 2A). The cumulative probability of a first relapse was 41.3% for POMS with HET, whereas it was 73.1% for those with MET at 5 years (Figure 2B). After adjustment for the treatment initiation epoch, the treatment group outcome persisted, and children beginning HET had an associated 54% lower risk of first relapse than those taking MET (adjusted hazard ratio [HR], 0.46; 95% CI, 0.31-0.67; *P* < .001) (eTables 5 and 6 in Supplement 1). The risk decreased by 7.0% by year of treatment initiation, meaning a higher efficacy of DMT in the latest epochs (adjusted HR, 0.93; 95% CI, 0.89-0.96; *P* < .001). Interestingly, neither age at DMT initiation nor age at disease onset emerged as confounders, and no interaction was observed.

HET was associated with a reduction in ARR by 91.6%, from 2.69 (95% CI, 2.31-3.12) to 0.23 (95% CI, 0.16-0.31) at 24 months. With a lesser magnitude, MET was associated with a decrease in ARR by 74.0%, from 1.93 (95% CI, 1.77-2.11) to 0.50 (95% CI, 0.45-0.55). A marked reduction in the relapse rate was observed to be associated with HET 3 to 6 months after treatment initiation (Figure 2C).

We confirmed these results using a conventional propensity score–weighted method. The confounder-adjusted (treatment initiation epoch) percentage of patients experiencing at least 1 relapse within the 5 years postinitiation was 44.8% and 72.4% in the HET and MET groups, respectively. The corresponding HR for POMS treated with HET vs MET was 0.41 (95% CI, 0.26-0.60; *P* < .001) (eTable 7 and eFigure 1 in Supplement 1).

To rule out potential treatment misclassification as HET or MET, we conducted a sensitivity analysis including only MS-approved DMT and rituximab (as anti-CD20 therapies are approved in adult MS). Findings were similar to those of the primary analysis (data not shown). Additionally, we conducted an analysis using an as-treated approach (ie, censoring data at treatment discontinuation), which demonstrated similar results to the intention-to-treat approach (adjusted HR, 0.41; 95% CI, 0.33-0.52; *P* < .001) (eFigure 2 and eTable 8 in Supplement 1).

### Brain MRI Disease Activity Over a 2-Year Period

After adjustment for confounding factors in a backward stepwise multivariate logistic regression, including EDSS and the number of T2 lesions at the index MRI, we found a 66% significant decrease in MRI activity at 2 years in the HET group compared with the MET group (n = 300; adjusted OR, 0.34; 95% CI, 0.18-0.66; *P* = .001) (eTables 9 and 10 in Supplement 1).

### Disability Assessment

The treatment strategy did not alter the CDP dynamics (available baseline EDSS: HET: n = 56; MET: n = 175). The annual probability of CDP gradually increased to reach 7% at 5 years after treatment initiation, and a cumulative probability of CDP of 16.1% was estimated regardless of group assignment (eTables 11 and 12 and eFigure 3 in Supplement 1).

Then, we analyzed the association of index treatment with tertiary education attainment (data available for 281 of 356 patients). Most children entered college of higher education or university regardless of initial treatment strategy (HET: 20 of 38 [52.6%]; MET: 160 of 243 [65.8%]) with an adjusted OR (HET vs MET) of 0.51 (95% CI, 0.24-1.10; *P* = .09) (eTable 13 in Supplement 1).

### Treatment Discontinuation and Switching

**Figure 3.**
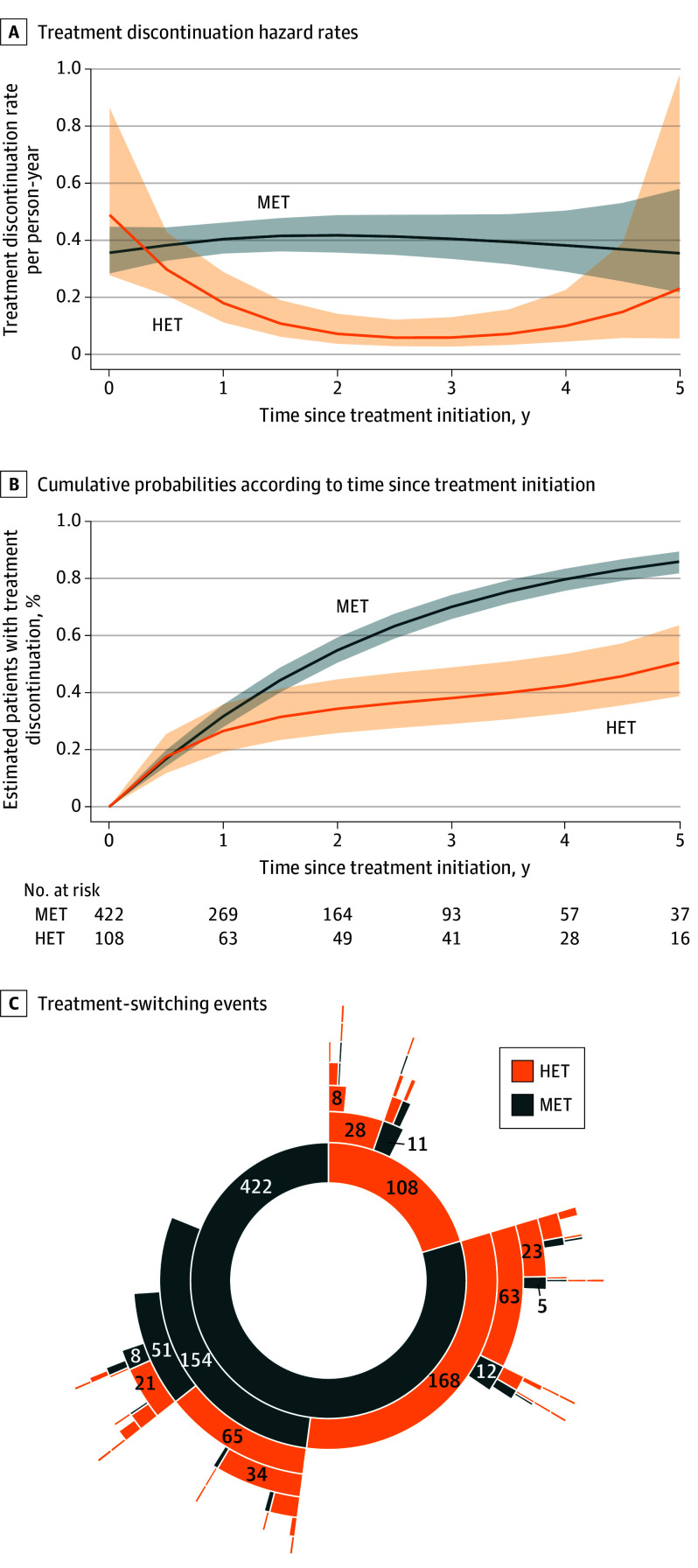
Treatment Discontinuation According to Index Treatment Strategy Group A, Treatment discontinuation hazard rates. B, Cumulative probabilities according to time since treatment initiation. C, Treatment-switching events depicted as a sunburst chart. Each ring symbolizes a treatment line. The inner ring contains the distribution of the initial group, with subthemes radiating out (ie, an outer ring illustrates a treatment switch from its corresponding inner ring). The size of each segment reflects the number of occurrences, and hence the relative importance of switches. Embedded numbers correspond to the number of patients in each subgroup. HET indicates highly effective therapy; MET, moderately effective therapy.

Regarding treatment discontinuation, the best-fitting model shows that the dynamic of treatment discontinuation rates were different between HET and MET, despite similar rates at baseline. Discontinuation rates of the MET group remained constant (40 withdrawals per 100 person-years), whereas those of the HET group decreased the first 2 years (7 withdrawals per 100 person-years) then increased (Figure 3A). The median (IQR) time to treatment discontinuation was 1.75 (1.55-1.95) years and 4.95 (3.25-5) years in the MET and HET groups, respectively. Overall, 50.7% of children taking HET had discontinued treatment at 5 years vs 86.1% of children taking MET (Figure 3B). The HR of MET discontinuation was 5.97 (95% CI, 2.92-12.20) after 2 years of treatment compared with that of HET, peaked around 2.5 years, and then decreased (eFigure 4 in Supplement 1). No confounders were found.

To pinpoint reasons for treatment discontinuation, the reported events of inefficacy and intolerance within 5 years of follow-up (eTable 2 in Supplement 1) and at last visit (Table 2) were analyzed. Unlike MET, few events were reported in the HET group (8 for inefficacy and 5 for intolerance) and were all observed within the first 6 months of treatment. Insufficient efficacy (HET: 13.6%; MET: 63.9%) (eFigure 5A in Supplement 1) and intolerance (HET: 6.6%; MET: 44.8%) (eFigure 5B in Supplement 1) predominated in the MET group. Most patients who initiated HET continued their treatment (64 of 108 [59.3%]), and among patients who underwent a first switch, 28 of 39 (71.8%) switched to another HET. In the MET group, only 86 of 422 children (20.4%) continued taking the treatment. One-half of the patients taking MET who switched were escalated to HET (168 of 322 [52.2%]) (Figure 3C and eTable 1 and eFigure 6 in Supplement 1). Thus, after first treatment switching, an HET strategy was considered in 92 of 108 children (85.2%) from the HET group, whereas 240 of 422 children (56.9%) from the MET group continued to take an initial MET, including horizontal switches. At the date of data extraction, 257 of 422 children (60.9%) taking an initial MET had escalated to an HET with a median (IQR) time to switch of 1.9 (0.9-3.8) years (Figure 3C and eFigure 6 in Supplement 1).

**Table 2.** Reasons for First Treatment Discontinuation During Follow-Up

Reason	No. (%)		
	Total	HET	MET
Discontinuation	380/530 (71.7)	44/108 (40.7)	336/422 (79.6)
SAE	12 (3.2)	3 (6.8)	9 (2.7)
Inefficacy	188 (49.5)	12 (27.3)	176 (52.4)
Intolerance (general, local, and/or biological)	112 (29.5)	5 (11.4)	107 (31.8)
Pregnancy (desire for)	8 (2.1)	2 (4.5)	6 (1.8)
Scheduled stop	36 (9.5)	21 (47.7)	15 (4.5)
Patient convenience	46 (12.1)	6 (13.6)	40 (11.9)
Other	9 (2.4)	3 (6.8)	6 (1.8)
Unknown	7 (1.8)	0	7 (2.1)

Abbreviations: HET, highly effective therapy; MET, moderately effective therapy; SAE, serious adverse event.

### SAEs

Among the 185 children who initiated their treatment after January 1, 2017, only 7 SAEs in 5 children were observed during the exposure period (4 in the HET group, 3.41 per 100 person-years vs 3 in the MET group, 1.67 per 100 person-years; *P* = .25) (eTable 14 in Supplement 1).

## Discussion

This French multicenter observational cohort study explored the effectiveness of the initial treatment strategy in POMS. Consistent with other registries, POMS prevalence was approximately 5.2% in the OFSEP cohort.^8,18^ The analysis showed that beginning an HET in children was associated with effectively controlled relapses and radiological activity compared with an MET. Treatments were initiated at a similar timing during the disease course, and both treatment strategies were associated with a significant reduction in relapses in the first 2 years. Early HET initiation was associated with optimally controlled POMS inflammatory activity, but MET posed a higher associated risk of DMT interruption and switching due to persistent disease activity and intolerance. HET interruption slightly increased beyond 3 years, explained by scheduled switches (due to anti–John Cunningham virus antibody positivity with natalizumab). The analysis herein highlights the scarcity of midterm SAEs, balanced between treatment groups over a 2-year period. Long-term safety is crucial, especially in young patients exposed to treatments during critical developmental periods (eg, neurodevelopment, puberty, risk of malignancies). Moreover, the potential safety profile heterogeneity of HET should be considered in decision-making.^36,37^

Few multicenter observational studies have compared initial DMT effectiveness in POMS. A large US study (197 patients taking newer DMTs and 544 taking injectable older DMTs) demonstrated better control of relapse and MRI activity with newer DMTs.^10,38^ However, most studies have compared injectable DMTs to newer DMTs, which combine both HET and MET, complicating preferred strategy evaluation.^9,10,13^ Similarly, the effectiveness assessment of DMT classified according to treatment efficacy in a small cohort study showed results similar to our study.^39^

Herein, DMF was classified among MET based on the most conservative assumption because no robust RCT has yet shown that DMF is more effective than platform therapies or teriflunomide, neither in adults nor children.^4,40,41^

Limiting long-term disability is critical, but no differences were found between strategies, possibly due to missing baseline EDSS scores and a relatively short follow-up duration, as in other studies.^10^ Yet, we cannot rule out that patients initially treated with MET who escalated to HET were ultimately sufficiently protected against EDSS progression at 5 years. A large Italian MS registry study highlighted long-term disability improvement in POMS in the most recent years, emphasizing increased HET use and earlier intervention as main progress in POMS management.^8^

Although personalized therapy would be a tremendous achievement in MS care, it would require proper identification of early prognostic factors, estimates of disease trajectory, and treatment-responsiveness profiling.^12,42^ In this regard, opting for an effective initial treatment strategy might limit disease breakthrough. In this cohort, inefficacy and intolerance were the main reasons for discontinuation, suggesting suboptimal strategies. As expected, disease breakthrough was observed mainly in the MET group with a rate of treatment escalation of approximately 60%, exceeding previous reports.^9,12,43,44^ Meanwhile, few patients taking HET underwent de-escalation during follow-up (<10%).

In adult MS, preferring HET as index treatment over therapeutic escalation was associated with a reduction in disease progression and long-term disability.^25,45,46,47^ Early treatment initiation within 2 years of disease onset can dampen disability progression also in children, highlighting an optimal time window to mitigate neurological damage.^15,48^

### Limitations

This study has some limitations. The main limitations include the observational nature of the study, lacking randomization. However, contrary to adult studies, such therapeutic strategy studies in children are hindered by ethical, practical, and demographic concerns; real-world observational studies are thus essential.^24,36,37,49,50^ The retrospective nature of the study, missing baseline data and heterogeneity in HET usage, were addressed by adjusting models. Similarly, the treatment switch rates observed might have evolved over time, leading to an underestimation of switching rates in older epochs. Although the classification of off-label therapies might be controversial, excluding them did not alter the primary outcome. Regarding disease breakthrough, an International Paediatric Multiple Sclerosis Study Group consensus defined treatment inefficacy. Yet, data on ineffectiveness collected in this registry were based on MS clinician judgment and not on predefined criteria.^21^ Treatment adherence was not specifically assessed herein but tolerability data were collected. Multiple confounders such as socioeconomic status and geographical origin could impact the educational course of children with POMS and partly explain the contrast between the present findings and those assessing the effects of natalizumab or fingolimod on cognition and quality of life.^2,51^ Additionally, missing demographic data including ethnicity (whose collection is banned by French law), socioeconomic status, access to an MS center, and other social determinants of health could affect the generalization of the present findings to a broader population.

## Conclusions

The findings of this cohort study suggest a sustained reduction in disease activity over 5 years associated with use of an HET as the primary strategy in POMS, with an optimal impact within the first 2 years. Although long-term safety studies are crucial, the apparent safety of MET is marred by treatment discontinuation and lesser early effect on disease control. The present findings corroborate current expert opinions and suggest prioritizing initial HET in children with POMS.^22,23,24,52^

## Notes

Supplement 1.
eMethods
**eTable 1.** Follow-Up Clinical Features **eTable 2.** Incidence Rates of Primary and Secondary Outcomes According to Follow-Up Duration **eTable 3.** Effect of Treatment **eTable 4.** Effect of Confounder Factor **eTable 5.** Unadjusted Hazard Ratio of Potential Confounding Factors Associated With the Occurrence of a First Relapse **eTable 6.** Adjusted Hazard Ratio for Covariates With Proportional and Linear Effects Associated With the Occurrence of a First Relapse **eTable 7.** Baseline Characteristics of Treatment Groups After Propensity Score Matching **eFigure 1.** Cumulative Hazard of First Relapse Event According to Time Since Treatment Initiation After Propensity Score Adjustment **eFigure 2.** Unadjusted Hazard Ratio of Potential Confounding Factors Associated With the Occurrence of a First Relapse **eTable 8.** Unadjusted Hazard Ratio of Potential Confounding Factors Associated With the Occurrence of a First Relapse in an As-Treated Approach **eTable 9.** Factors Associated With Brain MRI Disease Activity Status At 2 Years **eTable 10.** Adjusted Odds Ratio of Brain MRI Disease Activity at 2 Years (Multivariate Logistic Regression) **eTable 11.** Effect of Treatment **eFigure 3.** Dynamic and Cumulative Probability of EDSS Progression at 5 Years **eTable 12.** Unadjusted Hazard Ratio of Potential Confounding Factors Associated With Confirmed Disease Progression **eTable 13.** Adjusted Odds Ratio of High-Level Education Attainment **eFigure 4.** Hazard Ratio of HET Discontinuation Compared With MET Over Time **eFigure 5.** Five Years Cumulative Probability of Initial DMT Discontinuation Due to Inefficacy and Intolerance **eFigure 6.** Flow Chart of MET Discontinuation **eTable 14.** Period of Exposure After Treatment Withdrawal to Consider a Serious Adverse Event
eReferences

Supplement 2. **Nonauthor Collaborators.** OFSEP (Observatoire Français de la Sclérose en Plaques) Investigators.

Supplement 3.
Data Sharing Statement.
