# Supplementary material for: Highly Effective Therapies as First-Line Treatment for Pediatric-Onset Multiple Sclerosis
Source: JAMA Neurol. 2024 Feb 12;81(3):273–82. doi: 10.1001/jamaneurol.2023.5566 (PMC10862269; doi:10.1001/jamaneurol.2023.5566)
Supplement: Supplement 1. — eMethods eTable 1. Follow-Up Clinical Features eTable 2. Incidence Rates of Primary and Secondary Outcomes According to Follow-Up Duration eTable 3. Effect of Treatment eTable 4. Effect of Confounder Factor eTable 5. Unadjusted Hazard Ratio of Potential Confounding Factors Associated With the Occurrence of a First Relapse eTable 6. Adjusted Hazard Ratio for Covariates With Proportional and Linear Effects Associated With the Occurrence of a First Relapse eTable 7. Baseline Characteristics of Treatment Groups After Propensity Score Matching eFigure 1. Cumulative Hazard of First Relapse Event According to Time Since Treatment Initiation After Propensity Score Adjustment eFigure 2. Unadjusted Hazard Ratio of Potential Confounding Factors Associated With the Occurrence of a First Relapse eTable 8. Unadjusted Hazard Ratio of Potential Confounding Factors Associated With the Occurrence of a First Relapse in an As-Treated Approach eTable 9. Factors Associated With Brain MRI Disease Activity Status At 2 Years eTable 10. Adjusted Odds Ratio of Brain MRI Disease Activity at 2 Years (Multivariate Logistic Regression) eTable 11. Effect of Treatment eFigure 3. Dynamic and Cumulative Probability of EDSS Progression at 5 Years eTable 12. Unadjusted Hazard Ratio of Potential Confounding Factors Associated With Confirmed Disease Progression eTable 13. Adjusted Odds Ratio of High-Level Education Attainment eFigure 4. Hazard Ratio of HET Discontinuation Compared With MET Over Time eFigure 5. Five Years Cumulative Probability of Initial DMT Discontinuation Due to Inefficacy and Intolerance eFigure 6. Flow Chart of MET Discontinuation eTable 14. Period of Exposure After Treatment Withdrawal to Consider a Serious Adverse Event eReferences [file jamaneurol-e235566-s001.pdf]

Supplementary Online Content

Benallegue N, Rollot F, Wiertlewski S, et al; OFSEP (Observatoire Français de la Sclérose en Plaques) Investigators. Highly effective therapies as first-line treatment for pediatric-onset multiple sclerosis. *JAMA Neurol*. Published online February 12, 2024. doi:10.1001/jamaneurol.2023.5566

eMethods

eTable 1. Follow-Up Clinical Features

eTable 2. Incidence Rates of Primary and Secondary Outcomes According to Follow-Up Duration

eTable 3. Effect of Treatment

eTable 4. Effect of Confounder Factor

eTable 5. Unadjusted Hazard Ratio of Potential Confounding Factors Associated With the Occurrence of a First Relapse

eTable 6. Adjusted Hazard Ratio for Covariates With Proportional and Linear Effects Associated With the Occurrence of a First Relapse

eTable 7. Baseline Characteristics of Treatment Groups After Propensity Score Matching

eFigure 1. Cumulative Hazard of First Relapse Event According to Time Since Treatment Initiation After Propensity Score Adjustment

eFigure 2. Unadjusted Hazard Ratio of Potential Confounding Factors Associated With the Occurrence of a First Relapse

eTable 8. Unadjusted Hazard Ratio of Potential Confounding Factors Associated With the Occurrence of a First Relapse in an As-Treated Approach

eTable 9. Factors Associated With Brain MRI Disease Activity Status At 2 Years

eTable 10. Adjusted Odds Ratio of Brain MRI Disease Activity at 2 Years (Multivariate Logistic Regression)

eTable 11. Effect of Treatment

eFigure 3. Dynamic and Cumulative Probability of EDSS Progression at 5 Years

eTable 12. Unadjusted Hazard Ratio of Potential Confounding Factors Associated With Confirmed Disease Progression

eTable 13. Adjusted Odds Ratio of High-Level Education Attainment

eFigure 4. Hazard Ratio of HET Discontinuation Compared With MET Over Time

© 2024 Benallegue N et al. *JAMA Neurology.*

eFigure 5. Five Years Cumulative Probability of Initial DMT Discontinuation Due to Inefficacy and Intolerance

eFigure 6. Flow Chart of MET Discontinuation

eTable 14. Period of Exposure After Treatment Withdrawal to Consider a Serious Adverse Event

eReferences

This supplementary material has been provided by the authors to give readers additional information about their work.

© 2024 Benallegue N et al. *JAMA Neurology.*

eMethods

Patient inclusion criteria for secondary analyses

For analyses in which confirmed progression of disability was the studied outcome, two or more recorded disability scores at least 6 months apart were required after baseline and remote from any relapse (30 days) as well as a disability score at treatment initiation. For analyses of MRI disease activity, the availability of brain MRI data in the first 9 months as well as at 2 years was required.

Treatment procedures

Alemtuzumab was given intravenously for 5 days (cycle 1), then daily for 3 days (all subsequent cycles), fingolimod was oral once per day, mitoxantrone was intravenous every 3 months, natalizumab was intravenous every 4 weeks, ocrelizumab was intravenous every 24 weeks, ofatumumab was subcutaneous every 4 weeks, and rituximab was intravenous as one dose or two doses 2 weeks apart followed by one or two doses at intervals of 6-12 months thereafter.[1-5]

Outcomes

Relapses were collected by a neurologist with expertise in MS during patient clinical follow-up and entered on the EDMUS software. A relapse was defined as the appearance, recurrence, or worsening of neurological signs due to MS immediately preceded by a stable or improved neurological state lasting a least 30 days. Also, neurological symptoms had to persist at least 24h, without fever, and were accompanied by objective neurological aggravation different from fatigue alone.[6]

Statistical analyses

We used an innovative analytical method developed by the biostatistics department of the Hospices Civils de Lyon to model the logarithm of the event rates (ER) by a multidimensional penalized spline function based on tensor product splines. [7,8] This method allows to model the dynamic of ER (i.e. the evolution of the ER according to the follow-up) and the effects of covariates on this dynamic; this model is flexible, as the effects can be non-linear and/or time-dependent (i.e. non-proportional), meaning that the dynamic of ER may change smoothly with each covariate. Once estimated, the dynamic of ER can be represented graphically according to the follow-up. The ER is a fundamental concept but is not always easily interpretable (because it is a conditional probability per unit of time and can therefore be greater than one). However, when ER is low (<0·10), it may be easily translated in probability of event per unit of time; for example, a constant rate of 0·05 event per person-year over 1 year corresponds approximately to a probability of event of 5% within the year.

© 2024 Benallegue N et al. *JAMA Neurology.*

Supplementary: Outcomes

eTable 1: Follow-up clinical features.

	Total	HET	MET
**Children with POMS included**	530	108 (*20.4%*)	422 (*79.6%*)
**Age at the last visit**			
**Mean +/- std**	21.9 +/- 3.67	20.3 +/- 3.35	22.3 +/- 3.62
**Median [Q1-Q3]**	21.7 [19.1-24.7]	20.1 [18.0-22.1]	22.5 [19.5-25.1]
**(Min-Max)**	(11.4-30.0)	(11.6-28.7)	(11.4-30.0)
**Duration of follow-up (years)**			
**Mean +/- std**	5.8 +/- 3.56	4.0 +/- 3.11	6.3 +/- 3.5
**Median [Q321-Q3]**	5.8 [3.0-8.7]	3.4 [1.0-5.9]	6.6 [3.6-9.3]
**(Min-Max)**	(0-12.9)	(0-11.8)	(0-12.9)
**Duration of treatment (years)**			
**Mean +/- std**	2.1 +/- 2.03	2.4 +/- 2.38	2.1 +/- 1.93
**Median [Q1-Q3]**	1.4 [0.6-3.0]	1.4 [0.4-4.0]	1.5 [0.7-2.9]
**(Min-Max)**	(0-11.8)	(0-11.8)	(0-10.5)
**Percentage of follow-up time with treatment**			
**Mean +/- std**	91.5 +/- 16.77	92.6 +/- 17.52	91.2 +/- 16.62
**Median [Q1-Q3]**	98.7 [91.7-100]	100 [94.9-100]	98.2 [91.4-100]
**(Min-Max)**	(4.8-100)	(6.4-100)	(4.8-100)
**EDSS density [95% CI] (Number of EDSS scoring assessment per year)**	1.36 [1.14-1.61]	1.60 [1.36-1.85]	1.32 [1.12-1.57]
**Brain MRI density [95% CI] (Number of brain MRI per year)**	0.71 [0.56-0.90]	0.82 [0.66-1.02]	0.69 [0.55-0.88]
**Spinal cord MRI density [95% CI] (Number of spinal MRI per year)**	0.26 [0.17-0.38]	0.27 [0.18-0.39]	0.26 [0.18-0.38]
**Treatment discontinuation**			
**Yes**	380 (*71.7%*)	44 (*40.7%*)	336 (*79.6%*)
**No**	150 (*28.3%*)	64 (*59.3%*)	86 (*20.4%*)
**Treatment switching**			
**Yes**	361 (*68.1%*)	39 (*36.1%*)	322 (*76.3%*)
**No**	169 (*31.9%*)	69 (*63.9%*)	100 (*23.7%*)
**If yes, Type of DMT**			
**Azathioprine**	2 (*0.6%*)	0	2 (*0.6%*)
**Cladribine**	1 (*0.3%*)	0	1 (*0.3%*)
**Cyclophosphamide**	2 (*0.6%*)	2 (*5.1%*)	0
**Dimethyl Fumarate**	65 (*18.0%*)	1 (*2.6%*)	64 (*19.9%*)
**Fingolimod**	78 (*21.6%*)	8 (*20.5%*)	70 (*21.7%*)
**Glatiramer acetate**	19 (*5.3%*)	2 (*5.1%*)	17 (*5.3%*)
**Interferon beta 1a**	33 (*9.1%*)	4 (*10.3%*)	29 (*9.0%*)
**Interferon beta 1b**	2 (*0.6%*)	0	2 (*0.6%*)
**Mitoxantrone**	3 (*0.8%*)	2 (*5.1%*)	1 (*0.3%*)
**Natalizumab**	87 (*24.1%*)	4 (*10.3%*)	83 (*25.8%*)
**Ocrelizumab**	23 (*6.4%*)	11 (*28.2%*)	12 (*3.7%*)
**Peginterferon beta 1a**	9 (*2.5%*)	1 (*2.6%*)	8 (*2.5%*)
**Rituximab**	4 (*1.1%*)	3 (*7.7%*)	1 (*0.3%*)
**Teriflunomide**	31 (*8.6%*)	0	31 (*9.6%*)

© 2024 Benallegue N et al. *JAMA Neurology.*

**Blinded (clinical trials)**	2 (*0.6%*)	1 (*2.6%*)	1 (*0.3%*)
**Time to next treatment (months)**			
**Mean +/- std**	2.3 +/- 2.07	2.6 +/- 2.34	2.3 +/- 2.00
**Median [Q1-Q3]**	1.7 [0.7-3.2]	1.9 [0.5-4.1]	1.7 [0.8-3.0]
**(Min-Max)**	(0-11.8)	(0-11.8)	(0-10.5)
**Number of relapses within 24 months after baseline**			
**0**	283 (*53.4%*)	80 (*74.1%*)	203 (*48.1%*)
**1**	137 (*25.9%*)	18 (*16.7%*)	119 (*28.2%*)
**2**	68 (*12.8%*)	10 (*9.3%*)	58 (*13.7%*)
**3**	25 (*4.7%*)	0	25 (*5.9%*)
**≥ 4**	17 (*3.2%*)	0	17 (*4.0%*)

Abbreviations: CI: confidence interval; DMT: Disease-modifying treatments; EDSS: expanded disability status scale; HR: hazard ratio; HET: Highly-effective therapies; MET: Moderately-effective therapies; SAE: Serious adverse effects

eTable 2: Incidence rates of primary and secondary outcomes according to follow-up duration.

	HET		MET	
**Outcomes over** a **5-year period**	**N**	**Years of follow-up**	**N**	**Years of follow-up**
**First relapse**	31	244	272	828
**Brain MRI activity**	25	216	167	844
**Confirmed disability progression**	5	153	19	612
**Discontinuation of treatment**	40	232	316	799
**Discontinuation of treatment for inefficacy**	8	232	161	799
**Discontinuation of treatment for intolerance**	5	232	106	799

© 2024 Benallegue N et al. *JAMA Neurology.*

Supplementary: Model-building strategy for the primary analysis

eTable 3: Effect of treatment

In order to account for the effect of treatment, we propose to choose a model amongst the following candidate models:

Model	Formula $log[ER(t, treatment)] =$	Number of regression parameters	Number of smoothing parameters	EDF	AIC
M0	$f(t)$	6	1	2.9	1296.6
**M1**	$\boldsymbol{f(t) + treatment}$	**7**	**1**	**2.9**	**1270.0**
M2	$f(t) + treament + s(t) * HET$	12	2	2.0	1274.4

AIC: Akaike information criterion; EDF: effective degrees of freedom; ER: event rate (first relapse rate)

The keywords f and s stand for a one-dimensional penalised spline. The knot locations for time were fixed monthly.

<u>Interpretation</u>:

- M0: no effect of treatment
- M1: main effect of treatment without interaction with time; this model thus assumes that the effects of treatment are the same whatever the time values.
- M2: interaction between treatment and time, i.e the effect of treatment depends on time

Each model differs from the others in terms of numbers of regression and smoothing parameters. Because of penalisation, the number of regression parameters does not reflect the complexity of a model. For that purpose, we used the EDF instead. We chose a model amongst the candidate ones according to the AIC criterion corrected to take into account smoothing parameter uncertainty (Wood et al. 2016).

Thus, according to the AIC, we retained the M1 model.

eTable 4: Effect of confounder factor

In order to account for the year of treatment, we propose to choose a model amongst the following candidate models:

Model	Formula $log[ER(t, year)] =$	Number of regression parameters	Number of smoothing parameter s	EDF	AIC
M1	$f(t) + treatment$	7	1	2.9	1270.0
**M2**	$\boldsymbol{M1 + year}$	**13**	**1**	**3.1**	**1256.9**
M3	$M1 + s(year)$	24	2	3.1	1256.9
M4	$M1 + s(year) + s(year) * t$	134	3	8.5	1318.1
M5	$tensor(t, year) + group$	144	1	6.6	1257.4
M6	$M1 + treatment * s(year)$	36	3	3.1	1258.7

The keywords s and $tensor$ respectively stand for two one-dimensional penalised spline and a penalised tensor product spline. The knot locations for time and year were fixed every 6 months and yearly, respectively.

<u>Interpretation</u>:

- M1: model selected in the previous step
- M2: M1+ a linear and proportional effect of year
- M3: M1+ a non-linear and proportional effect of year
- M4: M1+ non-linear effect of year with interaction between linear effect of year and time; this model thus assumes that the effect of year depends on time.
- M5: tensor product between the year and time and a proportional effect of the treatment
- M6: M1+ a non-linear effect of year with interaction between the year and treatment

According to the AIC, we retained the M2 model.

Thus, the final model retained for the multivariate analysis was the one including linear and/or proportional effects of year and treatment.

© 2024 Benallegue N et al. *JAMA Neurology.*

References

Wood, S. N., Pya, N. and Säfken, B. (2016) Smoothing parameter and model selection for general smooth models. J. Am. Statist. Ass., 111, 1548-1563.

© 2024 Benallegue N et al. *JAMA Neurology.*

eTable 5: Unadjusted hazard ratio of potential confounding factors associated with the occurrence of a first relapse.

	Unadjusted HR	p-value
**Age at baseline [95% CI]**	0.99 [0.94-1.05]	0.83
**Treatment initiation epoch [95% CI]**		<0.001
**[2010 – 2012]**	1	
**[2013 – 2015]**	0.86 [0.66-1.11]	0.23
**[2016 - 2018]**	0.57 [0.42-0.78]	<0.001
**[2019 – 2022]**	0.46 [0.30-0.72]	<0.001
**EDSS baseline [95% CI]**		0.62
**0.0**	1	
**[0.5-3.5]**	1.00 [0.69-1.46]	0.99
**≥ 4.0**	0.75 [0.38-1.48]	0.41
**Unknown**	1.18 [0.87-1.60]	0.30
**Number of relapses the year before baseline [95% CI]**		0.45
**0**	1	
**1**	1.04 [0.53-2.04]	0.92
**2**	1.15 [0.58-2.28]	0.68
**≥ 3**	1.33 [0.66-2.70]	0.43
**Baseline brain T2 lesion load [95% CI]**		0.68
**0**	0.74 [0.10-5.29]	0.76
**< 9**	0.80 [0.53-1.20]	0.29
**≥ 9**	1	
**Not available**	1.09 [0.79-1.51]	0.60
**MRI scan not performed**	0.91 [0.70-1.18]	0.46

Abbreviations: CI: confidence interval; EDSS: expanded disability status scale; HR: hazard ratio; HET: Highly-effective therapies; MET: Moderately-effective therapies.

© 2024 Benallegue N et al. *JAMA Neurology.*

eTable 6: Adjusted hazard ratio for covariates with proportional and linear effects associated with the occurrence of a first relapse.

		Adjusted HR	p-value
**Year of treatment [95% CI]**		0.93 [0.89-0.96]	<0.001
**Treatment group [95% CI]**			
	**MET**	1	
	**HET**	0.46 [0.31-0.67]	<0.001

Abbreviations: CI: confidence interval; HR: hazard ratio; HET: Highly-effective therapies; MET: Moderately-effective therapies.

© 2024 Benallegue N et al. *JAMA Neurology.*

Supplementary: Sensitivity analysis using propensity scores by inverse probability of treatment weighting.

To validate our model-building strategy we used a more conventional statistical method, i.e. propensity scores by inverse probability of treatment weighting (IPTW) to compare the effectiveness of HET and MET on the occurrence of the first relapse in a sensitivity analysis.

eTable 7: Baseline characteristics of treatment groups after propensity score matching.

Variable	HET	MET	d Cohen*
**Treatment initiation epoch (%)**			<0.001
**[2010 – 2012]**	30.6	30.6	
**[2013 – 2015]**	27.5	27.5	
**[2016 - 2018]**	22.6	22.6	
**[2019 – 2022]**	19.2	19.2	

*Standardised mean or proportion difference (Cohen's d values): a value less than 0.2 is considered acceptable, between 0.2 and 0.5 considered as a moderate difference, between 0.5 and 0.8 as significant differences and greater than 0.8 as a major difference.

© 2024 Benallegue N et al. *JAMA Neurology.*

eFigure 1: Cumulative hazard of first relapse event according to time since treatment initiation after propensity score adjustment.

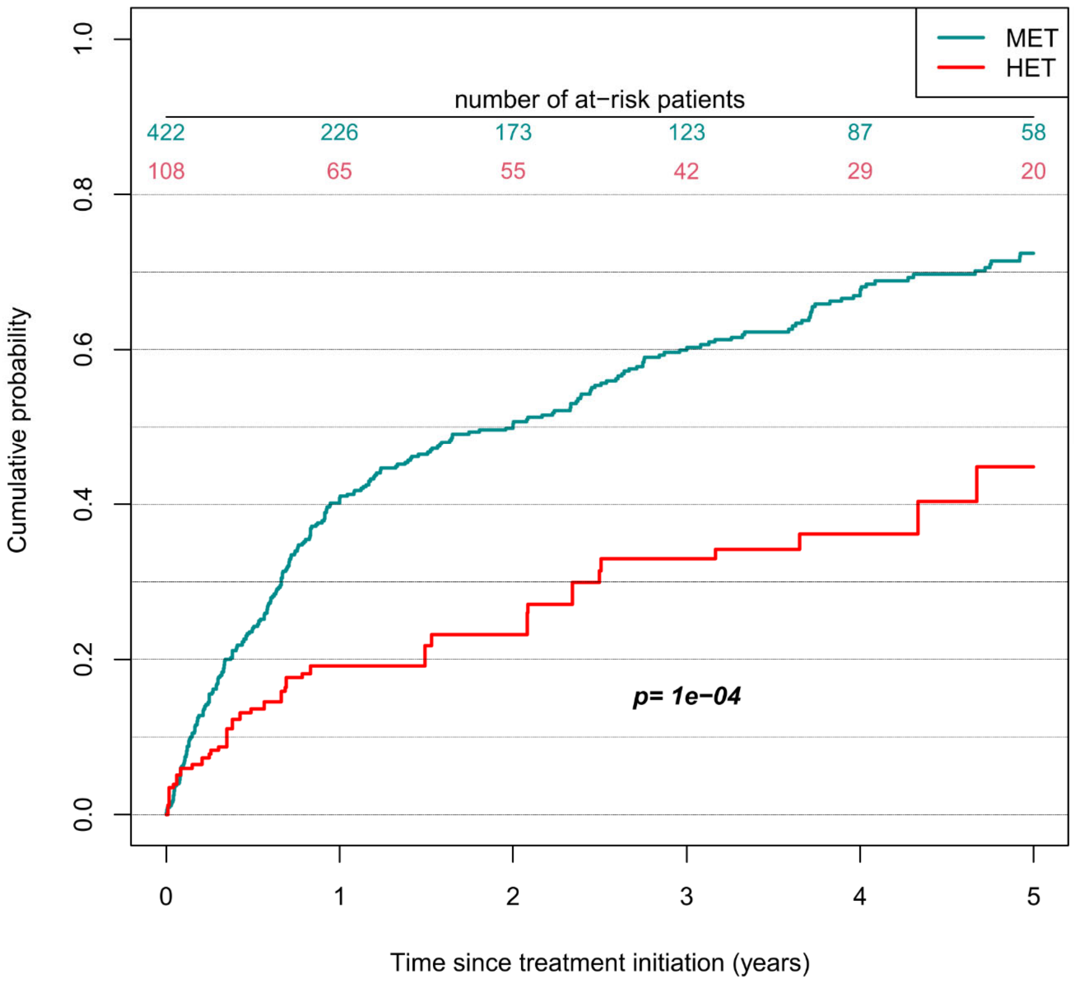

Using a conventional causal approach with propensity scores by inverse probability of treatment weighting (IPTW), we validated the results from the conditional approach with multivariate modelling. We observed a 59% reduction of the occurrence of a first relapse in the HET group compared to the MET group.

© 2024 Benallegue N et al. *JAMA Neurology.*

Supplementary: Sensitivity analysis using an as-treated approach for the primary outcome

eFigure 2: Unadjusted hazard ratio of potential confounding factors associated with the occurrence of a first relapse.

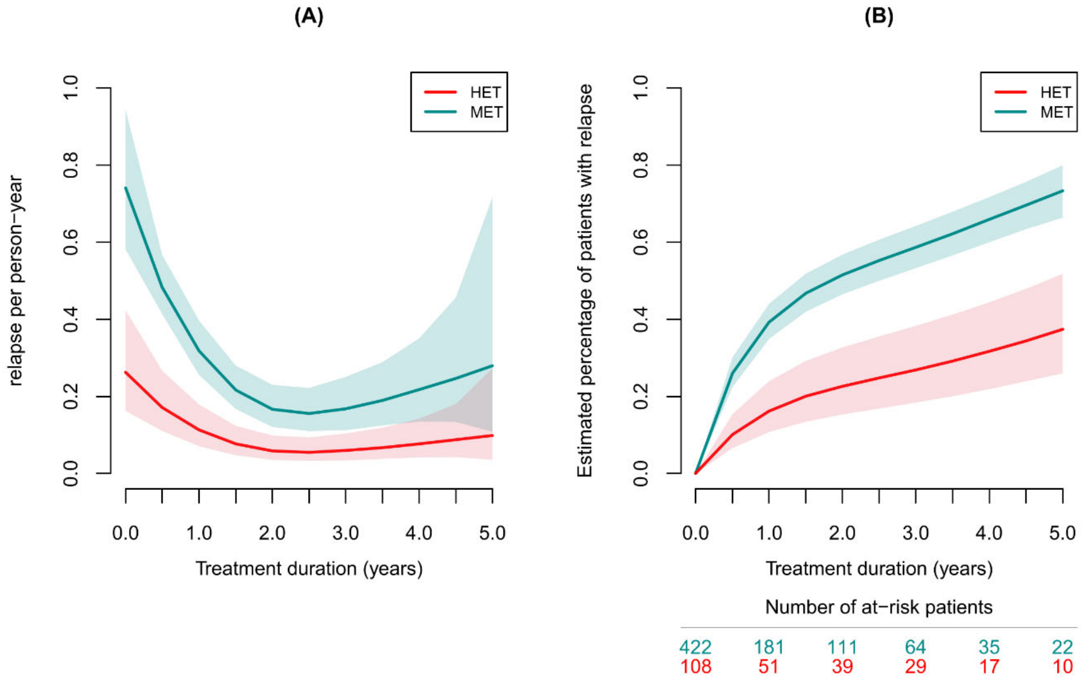

Relapse rate according to index treatment strategy using an as-treated analysis

(A) Time-to-first-relapse depicted as a dynamic of event rate and (B) cumulative probabilities of a first relapse occurrence after treatment initiation according to index DMT (HET=red; MET=blue).

© 2024 Benallegue N et al. *JAMA Neurology.*

eTable 8: Unadjusted hazard ratio of potential confounding factors associated with the occurrence of a first relapse in an as-treated approach.

	Unadjusted HR	p-value
**Age at baseline [95% CI]**	0.97 [0.91-1.03]	0.25
**Treatment initiation epoch [95% CI]**		<0.001
**[2010 – 2012]**	1	
**[2013 – 2015]**	0.80 [0.58-1.07]	0.13
**[2016 - 2018]**	0.57 [0.40-0.80]	<0.001
**[2019 – 2022]**	0.40 [0.24-0.65]	<0.001
**EDSS baseline [95% CI]**		0.21
**0.0**	1	
**[0.5-3.5]**	1.07 [0.68-1.66]	0.78
**≥ 4.0**	0.72 [0.26-2.02]	0.53
**Unknown**	1.32 [0.92-1.89]	0.13
**Number of relapses the year before baseline [95% CI]**		0.11
**0**	1	
**1**	2.22 [0.70-7.02]	0.17
**2**	2.63 [0.83-8.30]	0.10
**≥ 3**	2.94 [0.91-9.52]	0.07
**Baseline brain T2 lesion load [95% CI]**		0.75
**0**	1.12 [0.16-8.00]	0.91
**< 9**	0.73 [0.45-1.17]	0.19
**≥ 9**	1	
**Not available**	0.97 [0.65-1.43]	0.86
**MRI scan not performed**	0.91 [0.68-1.23]	0.55

© 2024 Benallegue N et al. *JAMA Neurology.*

Supplementary: Brain MRI disease activity over a 2-year period

eTable 9: Factors associated with brain MRI disease activity status at 2 years.

	MRI disease activity		
	**Yes**	**No**	**p-value**
**Treatment group**			0.002
**HET**	25 (*13%*)	30 (*27.8%*)	
**MET**	167 (*87%*)	78 (*72.2%*)	
**Age at baseline**			0.619
**< 10**	5 *(2.6%)*	1 *(0.9%)*	
**[10-12[**	8 *(4.2%)*	2 *(1.9%)*	
**[12-14[**	16 *(8.3%)*	10 *(9.3%)*	
**[14-16[**	47 *(24.5%)*	31 *(28.7%)*	
**[16-18[**	116 *(60.4%)*	64 *(59.3%)*	
**Treatment initiation epoch**			0.266
**[2010 – 2012]**	64 *(33.3%)*	26 *(24.1%)*	
**[2013 – 2015]**	53 *(27.6%)*	40 *(37.0%)*	
**[2016 - 2018]**	55 *(28.7%)*	31 *(28.7%)*	
**[2019 – 2022]**	20 *(10.4%)*	11 *(10.2%)*	
**EDSS (+/- 3 months)**			0.042
**0.0**	35 *(18.2%)*	24 *(22.2%)*	
**[0.5-3.5]**	40 *(20.8%)*	29 *(26.9%)*	
**≥ 4.0**	5 *(2.6%)*	8 *(7.4%)*	
**Not available**	112 *(58.3%)*	47 *(43.5%)*	
**Number of relapses in the year before baseline**			0.156
**1**	99 (51.6%)	47 (43.5%)	
**2**	69 (35.9%)	39 (36.1%)	
**3**	24 (12.5%)	22 (20.4%)	
**Brain MRI T2 lesion load (number of lesions)**			0.006
**0**	0	1 (*0.9%)*	
**< 9**	7 (*3.7%)*	14 (*13.0%)*	
**≥ 9**	156 (*81.3%)*	73 (*67.6%)*	
**not available**	29 (*15.1%)*	20 (*18.5%)*	

© 2024 Benallegue N et al. *JAMA Neurology.*

eTable 10: Adjusted odds ratio of brain MRI disease activity at 2 years (multivariate logistic regression).

	Adjusted OR	p-value
**Treatment group [95% CI]**		
**HET**	0.34 [0.18-0.66]	0.001
**MET**	1	
**Baseline EDSS [95% CI]**		0.045
**0.0**	1	
**[0.5-3.5]**	1.12 [0.53-2.36]	0.78
**≥ 4.0**	0.70 [0.19-2.66]	0.60
**Unknown**	2.09 [1.07-4.05]	0.030
**Brain MRI T2 lesion load (number of lesions) [95% CI]**		<0.001
**< 9**	1	
**≥ 9**	5.70 [2.15-15.13]	<0.001
**not available**	2.92 [0.98-8.70]	0.054

© 2024 Benallegue N et al. *JAMA Neurology.*

Supplementary: Disability progression

eTable 11: Effect of treatment

In order to account for the effect of treatment, we propose to choose a model amongst the following candidate models.

Model	Formula $\boldsymbol{log[\lambda(t, groupe)]} =$	Number of regression parameters	Number of smoothing parameters	EDF	AIC corrected
**M0**	$\boldsymbol{f(t)}$	**6**	**1**	**2.1**	**212.2**
M1	$f(t) + groupe$	7	1	3.1	214.2
M2	$f(t) + groupe + s(t) * HET$	12	2	4.3	214.4

AIC: Akaike information criterion; EDF: effective degrees of freedom; ER: event rate (confirmed disease progression rate)

The keywords f and s stand for a one-dimensional penalized spline. The knots locations for time were fixed monthly.

Interpretation:

- M0: no effect of treatment
- M1: main effect of treatment without interaction with time; this model thus assumes that the effects of treatment is the same whatever time values.
- M2: interaction between treatment and time i.e the effect of treatment depends on time

Every model differs from the others in terms of numbers of regression and smoothing parameters. Because of penalization, the number of regression parameters do not reflect the complexity of a model. For that purpose we use the EDF instead. We chose a model amongst the candidate ones according to the AIC criterion corrected to take into account smoothing parameter uncertainty (Wood et al. 2016).

Thus, according to AIC, we retained the M0 model without any effect of treatment group assignation.

eFigure 3: Dynamic and cumulative probability of EDSS progression at 5 years.

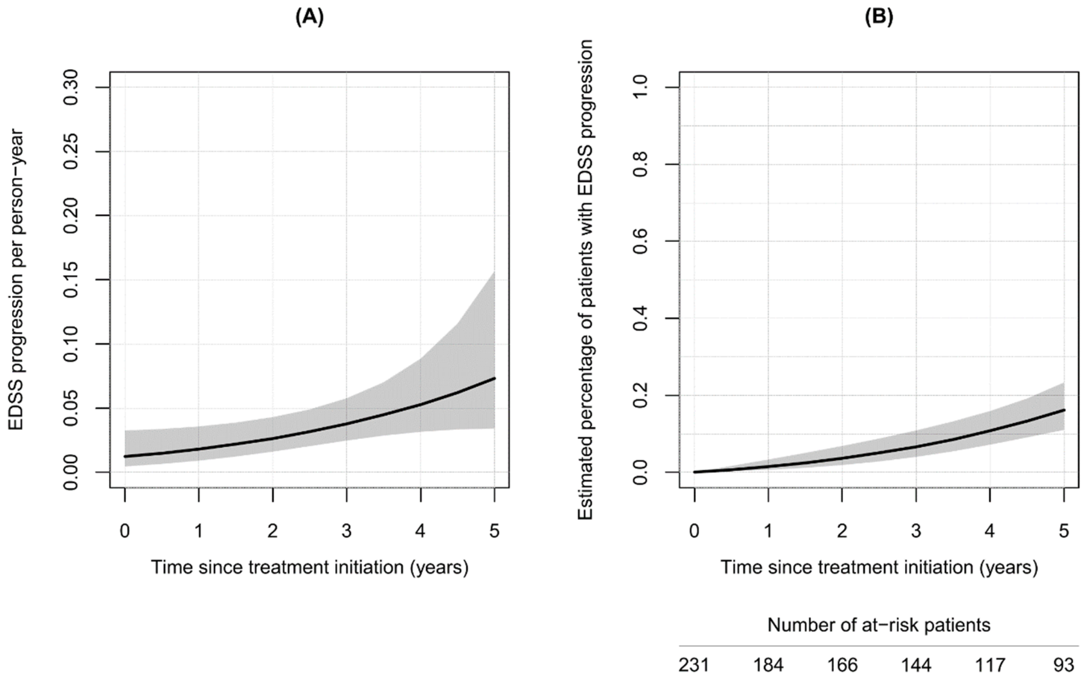

(A) Hazard rate and (B) cumulative probability of confirmed disability progression for both HET and MET groups 5 years after treatment initiation.

© 2024 Benallegue N et al. *JAMA Neurology.*

eTable 12 Unadjusted hazard ratio of potential confounding factors associated with confirmed disease progression.

	Unadjusted HR	p-value
**Age at baseline [95% CI]**	1.14 [0.93-1.40]	0.20
**Treatment initiation epoch [95% CI]**		0.96
**[2010 – 2012]**	1	
**[2013 – 2015]**	1.22 [0.56-2.66]	0.62
**[2016 - 2018]**	1.19 [0.47-3.03]	0.71
**[2019 – 2022]**	1.07 [0.12-9.06]	0.95
**Baseline EDSS [95% CI]**		0.99
**0.0**	1	
**[0.5-3.5]**	0.98 [0.48-2.02]	0.98
**≥ 4.0**	0.97 [0.31-2.97]	0.95
**Number of relapses the year before baseline [95% CI]**		0.50
**1**	1	
**2**	0.63 [0.29-1.38]	0.25
**≥ 3**	0.80 [0.33-1.92]	0.62
**Brain MRI T2 lesion load (number of lesions) [95% CI]**		0.33
**< 9**	0.56 [0.17-1.85]	0.34
**≥ 9**	1	
**not available**	1.67 [0.64-4.40]	0.30
**MRI scan not performed**	0.56 [0.17-1.86]	0.34

© 2024 Benallegue N et al. *JAMA Neurology.*

eTable 13: Adjusted odds ratio of high-level education attainment.

N=170		Adjusted OR	p-value
**Treatment group [95% CI]**			
	**HET**	1	
	**MET**	0.48 [0.17-1.39]	0.18
**Age at baseline [95% CI]**		0.90 [0.67-1.22]	0.51
**Treatment initiation epoch [95% CI]**			
	**[2010 – 2012]**	1	
	**[2013 – 2015]**	0.87 [0.38-1.98]	0.75
	**[2016 - 2022]**	1.37 [0.44-4.24]	0.59
**Baseline EDSS [95% CI]**			
	**0.0**	1	
	**[0.5-3.5]**	1.33 [0.36-4.92]	0.67
	**≥ 4.0**	1.08 [0.15-7.75]	0.94
	**Unknown**	1.22 [0.42-3.59]	0.72
**Number of relapses the year before baseline [95% CI]**			
	**0**	1	
	**1**	0.31 [0.03-3.43]	0.34
	**2**	0.46 [0.04-5.30]	0.53
	**≥ 3**	0.47 [0.04-5.77]	0.56
**Brain MRI T2 lesion load (number of lesions) [95% CI]**			
	**< 9**	1.68 [0.42-6.75]	0.46
	**≥ 9**	1	
	**not available**	0.92 [0.30-2.82]	0.88
	**MRI scan not performed**	0.90 [0.35-2.34]	0.83

© 2024 Benallegue N et al. *JAMA Neurology.*

Supplementary: Treatment discontinuation

eFigure 4: Hazard ratio of HET discontinuation compared with MET over time.

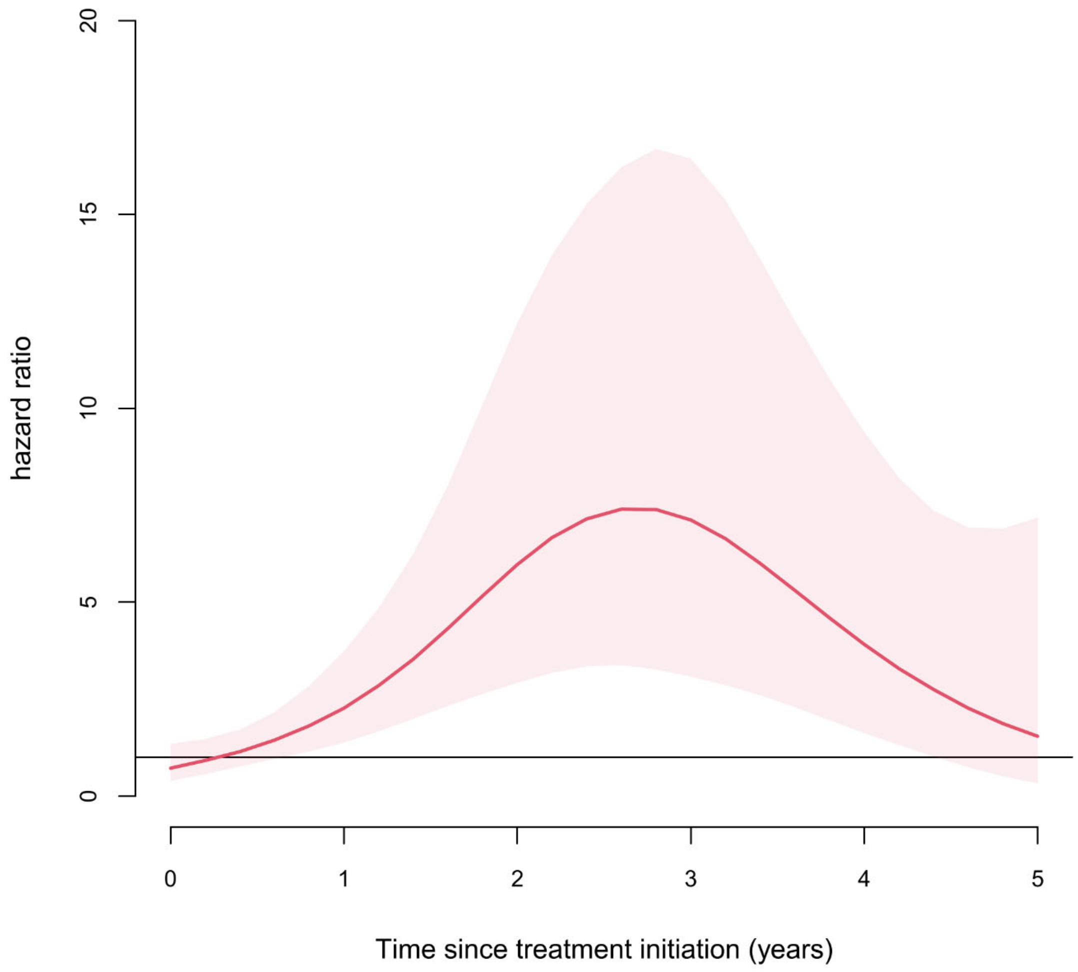

© 2024 Benallegue N et al. *JAMA Neurology.*

eFigure 5: Five-years cumulative probability of initial DMT discontinuation due to inefficacy and intolerance.

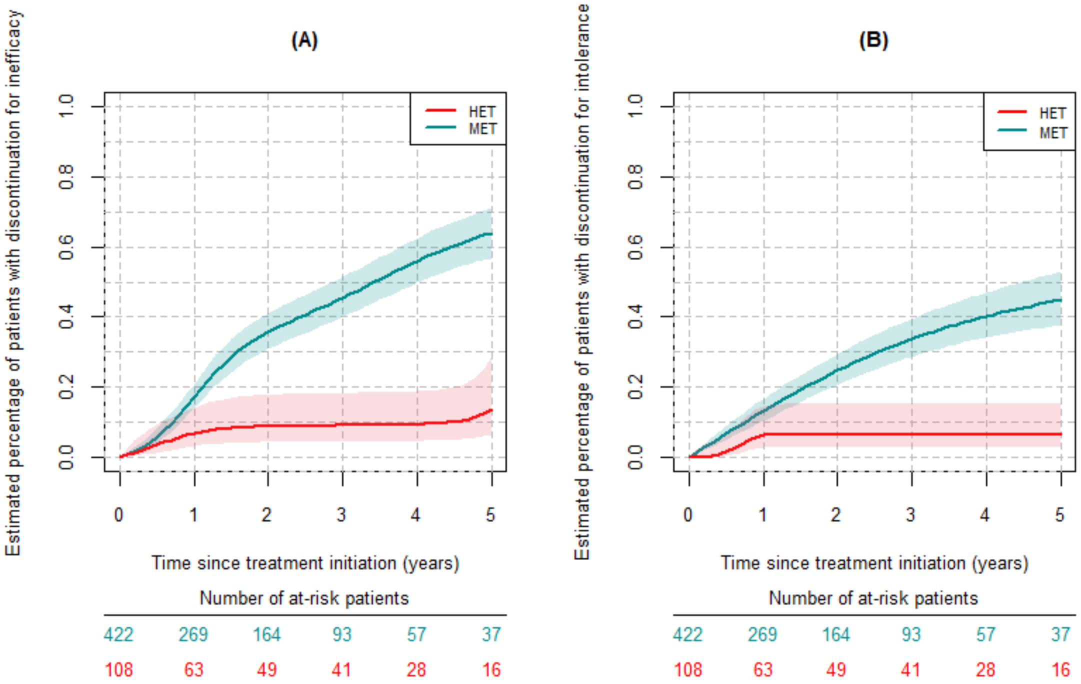

Cumulative probability of treatment discontinuation due to (A) inefficacy and (B) intolerance for both HET and MET groups 5 years after treatment initiation.

© 2024 Benallegue N et al. *JAMA Neurology.*

eFigure 6: Flow chart of MET discontinuation.

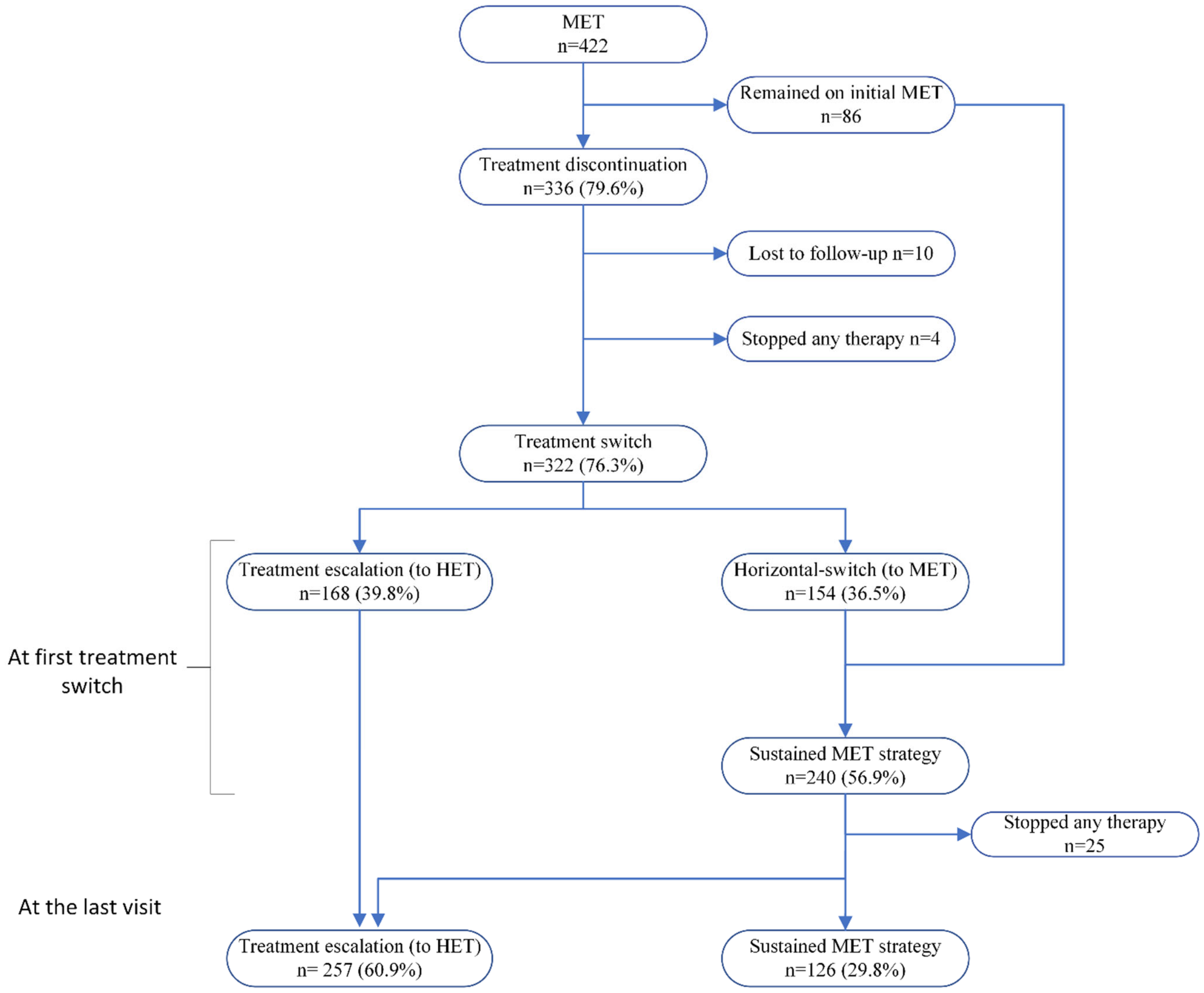

© 2024 Benallegue N et al. *JAMA Neurology.*

eTable 14: Period of exposure after treatment withdrawal to consider a serious adverse event.

DMT	Primary Risk Window (days)
Alemtuzumab	*364*
Azathioprine	*91*
Cladribine	*364*
Cyclophosphamide	*91*
Dimethyl fumarate	*91*
Fingolimod	*91*
Glatiramer acetate	*91*
Interferon β-1b	*91*
Interferon β-1a	*91*
Mitoxantrone	*91*
Mycophenolate Mofetil	*91*
Natalizumab	*91*
Ocrelizumab	*182*
Ofatumumab	*182*
Peginterferon β-1a	*91*
Rituximab	*182*
Teriflunomide	*121*

eReferences

1. Cohen JA, Coles AJ, Arnold DL, et al. Alemtuzumab versus interferon beta 1a as first-line treatment for patients with relapsing-remitting multiple sclerosis: a randomised controlled phase 3 trial. *Lancet* 2012; **380**(9856): 1819-28.
2. Edan G, Comi G, Le Page E, et al. Mitoxantrone prior to interferon beta-1b in aggressive relapsing multiple sclerosis: a 3-year randomised trial. *J Neurol Neurosurg Psychiatry* 2011; **82**(12): 1344-50.
3. Hauser SL, Bar-Or A, Comi G, et al. Ocrelizumab versus Interferon Beta-1a in Relapsing Multiple Sclerosis. *N Engl J Med* 2017; **376**(3): 221-34.
4. Gartner J, Hauser SL, Bar-Or A, et al. Efficacy and safety of ofatumumab in recently diagnosed, treatment-naive patients with multiple sclerosis: Results from ASCLEPIOS I and II. *Mult Scler* 2022; **28**(10): 1562-75.
5. Spelman T, Frisell T, Piehl F, Hillert J. Comparative effectiveness of rituximab relative to IFN-beta or glatiramer acetate in relapsing-remitting MS from the Swedish MS registry. *Mult Scler* 2018; **24**(8): 1087-95.
6. Schumacher GA, Beebe G, Kibler RF, et al. Problems of Experimental Trials of Therapy in Multiple Sclerosis: Report by the Panel on the Evaluation of Experimental Trials of Therapy in Multiple Sclerosis. *Ann N Y Acad Sci* 1965; **122**: 552-68.

© 2024 Benallegue N et al. *JAMA Neurology.*

7. Remontet L, Uhry Z, Bossard N, et al. Flexible and structured survival model for a simultaneous estimation of non-linear and non-proportional effects and complex interactions between continuous variables: Performance of this multidimensional penalized spline approach in net survival trend analysis. *Stat Methods Med Res* 2019; **28**(8): 2368-84.
8. Fauvernier M, Roche L, Uhry Z, et al. Multi-dimensional penalized hazard model with continuous covariates: applications for studying trends and social inequalities in cancer survival. *Journal of the Royal Statistical Society Series C: Applied Statistics* 2019; **68**(5): 1233-57.

© 2024 Benallegue N et al. *JAMA Neurology.*
