# Supplementary material for: Highly Effective Therapies as First-Line Treatment for Pediatric-Onset Multiple Sclerosis
Source: JAMA Neurol. 2024 Feb 12;81(3):273–82. doi: 10.1001/jamaneurol.2023.5566 (PMC10862269; doi:10.1001/jamaneurol.2023.5566)
Supplement: Supplement 2. — Nonauthor Collaborators. OFSEP (Observatoire Français de la Sclérose en Plaques) Investigators. [file jamaneurol-e235566-s002.pdf]

Supplemental Online Content: Nonauthor Collaborators
*First name, last name, and suffix (if applicable) are required and will appear in PubMed.

*Group Name(s): OFSEP (Observatoire Français de la Sclérose en Plaques) Investigators							
***First Name and Middle Initial(s)**	***Last Name**	***Suffix (eg, Jr, III)**	Academic Degrees	Institution	Location (city, state/province, country)	Role or Contribution, eg, chair, principal investigator	Group (if more than 1 Group listed in the byline) and/or Subgroup (eg, Steering Committee)
Cotton	François		MD	Hospices civils de Lyon, Hôpital Lyon sud, Service d'imagerie médicale et interventionnelle	Lyon/Pierre-Bénite, France	Steering Committee member	Steering committee
Douek	Pascal		MD	Union pour la lutte contre la sclérose en plaques (UNISEP)	Ivry-sur-Seine, France	Steering Committee member	Steering committee
Pachot	Alexandre		PhD	Hospices civils de Lyon, Direction de la Recherche en Santé	Lyon, France	Steering Committee member	Steering committee
Olaiz	Javier		PhD	Université Claude Bernard Lyon 1, Lyon ingéniérie projets	Lyon, France	Steering Committee member	Steering committee
Rigaud-Bully	Claire		N/A	Fondation Eugène Devic EDMUS contre la sclérose en plaques	Lyon, France	Steering Committee member	Steering committee
Marignier	Romain		MD	Hospices civils de Lyon, Hôpital Pierre Wertheimer, Service de neurologie A	Lyon/Bron, France	Investigator	Investigators
Le Page	Emmanuelle		MD	Centre hospitalier universitaire de Rennes, Hôpital Pontchaillou, Service de neurologie	Rennes, France	Investigator	Investigators
Collongues	Nicolas		MD	Hôpitaux universitaire de Strasbourg, Hôpital de Hautepierre, Service des maladies inflammatoires du système nerveux – neurologie	Strasbourg, France	Investigator	Investigators

Supplemental Online Content: Nonauthor Collaborators
*First name, last name, and suffix (if applicable) are required and will appear in PubMed.

***First Name and Middle Initial(s)**	***Last Name**	***Suffix (eg, Jr, III)**	Academic Degrees	Institution	Location (city, state/province, country)	Role or Contribution, eg, chair, principal investigator	Group (if more than 1 Group listed in the byline) and/or Subgroup (eg, Steering Committee)
Cohen	Mikaël		MD	Centre hospitalier universitaire de Nice, Université Nice Côte d'Azur, Hôpital Pasteur, Service de neurologie	Nice, France	Investigator	Investigators
Fromont	Agnès		MD	Centre hospitalier universitaire Dijon Bourgogne, Hôpital François Mitterrand, Service de neurologie, maladies inflammatoires du système nerveux et neurologie générale	Dijon, France	Investigator	Investigators
Audoin	Bertrand		MD	Assistance publique des hôpitaux de Marseille, Centre hospitalier de la Timone, Service de neurologie et unité neuro-vasculaire	Marseille, France	Investigator	Investigators
Giannesini	Claire		MD	Assistance publique des hôpitaux de Paris, Hôpital Saint-Antoine, Service de neurologie	Paris, France	Investigator	Investigators
Gout	Olivier		MD	Fondation Adolphe de Rothschild de l'œil et du cerveau, Service de neurologie	Paris, France	Investigator	Investigators
Camdessanché	Jean-Philippe		MD	Centre hospitalier universitaire de Saint-Étienne, Hôpital Nord, Service de neurologie	Saint-Étienne, France	Investigator	Investigators
Moulin	Solène		MD	Centre hospitalier universitaire de Reims, Hôpital Maison-Blanche, Service de neurologie	Reims, France	Investigator	Investigators
Doghri	Ines		MD	Centre hospitalier régional universitaire de Tours, Hôpital Bretonneau, Service de neurologie	Tours, France	Investigator	Investigators

Supplemental Online Content: Nonauthor Collaborators
*First name, last name, and suffix (if applicable) are required and will appear in PubMed.

***First Name and Middle Initial(s)**	***Last Name**	***Suffix (eg, Jr, III)**	Academic Degrees	Institution	Location (city, state/province, country)	Role or Contribution, eg, chair, principal investigator	Group (if more than 1 Group listed in the byline) and/or Subgroup (eg, Steering Committee)
Ben Nasr	Haifa		MD	Centre hospitalier sud francilien, Service de neurologie	Corbeil-Essonnes, France	Investigator	Investigators
Hankiewicz	Karolina		MD	Centre hospitalier de Saint-Denis, Hôpital Casanova, Service de neurologie	Saint-Denis, France	Investigator	Investigators
Pottier	Corinne		MD	Centre hospitalier de Pontoise, Service de neurologie	Pontoise, France	Investigator	Investigators
Neau	Jean-Philippe		MD	Centre hospitalier universitaire de Poitiers, Site de la Milétrie, Service de neurologie	Poitiers, France	Investigator	Investigators
Labeyrie	Céline		MD	Assistance publique des hôpitaux de Paris, Hôpital Bicêtre, Service de neurologie	Le Kremlin-Bicêtre, France	Investigator	Investigators
Nifle	Chantal		MD	Centre hospitalier de Versailles, Hôpital André-Mignot, Service de neurologie	Le Chesnay, France	Investigator	Investigators
