# Supplementary material for: Highly Effective Therapies as First-Line Treatment for Pediatric-Onset Multiple Sclerosis
Source: JAMA Neurol. 2024 Feb 12;81(3):273–82. doi: 10.1001/jamaneurol.2023.5566 (PMC10862269; doi:10.1001/jamaneurol.2023.5566)
Supplement: Supplement 3. — Data Sharing Statement. [file jamaneurol-e235566-s003.pdf]

Data Sharing Statement

Benallegue. Highly Effective Therapies as First-Line Treatment for Pediatric-Onset Multiple Sclerosis. *JAMA Neurol*. Published February 12, 2024. doi:10.1001/jamaneurol.2023.5566

Data
Data available: Yes
Data types: Deidentified participant data
How to access data: Anonymized data can be made available upon reasonable request to the corresponding author.
When available: With publication

Supporting Documents
Document types: None

Additional Information
Who can access the data: Data will be made available for researchers whose proposed use of the data has been approved by the corresponding author, the OFSEP scientific committee and the OFSEP steering committee.
Types of analyses: Anonymized data
Mechanisms of data availability: after approval of a proposal
